# Trehalose-6-Phosphate Synthase Contributes to Rapid Cold Hardening in the Invasive Insect *Lissorhoptrus oryzophilus* (Coleoptera: Curculionidae) by Regulating Trehalose Metabolism

**DOI:** 10.3390/insects14120903

**Published:** 2023-11-23

**Authors:** Juhong Zhang, Lizhong Qi, Baoyu Chen, Hongye Li, Lianglin Hu, Qingtai Wang, Shang Wang, Jinghui Xi

**Affiliations:** 1 College of Plant Science, Jilin University, Changchun 130062, China; zhjhqhd@126.com (J.Z.); qlz18795119237@163.com (L.Q.); hongye22@mails.jlu.edu.cn (H.L.); hull23@mails.jlu.edu.cn (L.H.); wangqt0201@126.com (Q.W.); shangwang@jlu.edu.cn (S.W.); 2 Key Laboratory of Plant Nutrition and Agro-Environment in Northeast Region, Ministry of Agriculture and Rural Affairs of the People’s Republic of China, Institute of Agricultural Resources and Environment Research, Jilin Academy of Agricultural Sciences, Changchun 130033, China; bych76@126.com

**Keywords:** trehalose-6-phosphate synthase, rapid cold hardening, trehalose metabolism, cold resistance, rice water weevil

## Abstract

**Simple Summary:**

Trehalose plays crucial roles in the cold tolerance of insects. Trehalose-6-phosphate synthase (TPS) is an important enzyme involved in trehalose synthesis. *TPS* genes have been demonstrated to be involved in cold-resistant physiological processes. *Lissorhoptrus oryzophilus* is an important invasive pest of rice in China. To gain insight into the function of TPS in rapid cold hardening (RCH), we cloned and characterized the *TPS* gene of *L. oryzophilus* (*LoTPS*). Next, we examined changes in gene expression and trehalose levels under low temperature after RCH treatment. We found that *LoTPS* was a fused gene with conserved TPS and trehalose-6-phosphate phosphatase (TPP) domains. As expected, RCH increased the survival rate of *L. oryzophilus* adults under low temperature. Furthermore, it led to an increase in *TPS* gene expression and trehalose content. However, RCH efficiency disappeared when the *LoTPS* gene was RNA-interfered, causing no significant increase in the survival rate, *TPS* gene expression or trehalose level. This study demonstrates the importance of TPS in enhancing cold tolerance through RCH. The *TPS* gene regulates trehalose synthesis and accumulation in adults, thus improving their survival under low temperatures. Our findings contribute to a better understanding of the cold tolerance mechanisms and invasiveness of *L. oryzophilus*.

**Abstract:**

Rapid cold hardening (RCH) is known to rapidly enhance the cold tolerance of insects. Trehalose has been demonstrated to be a cryoprotectant in *Lissorhoptrus oryzophilus*, an important invasive pest of rice in China. Trehalose synthesis mainly occurs through the Trehalose-6-phosphate synthase (TPS)/trehalose-6-phosphate phosphatase (TPP) pathway in insects. In this study, the *TPS* gene from *L. oryzophilus* (*LoTPS*) was cloned and characterized for the first time. Its expression and trehalose content changes elicited by RCH were investigated. Our results revealed that RCH not only increased the survival rate of adults but also upregulated the expression level of *LoTPS* and increased the trehalose content under low temperature. We hypothesized that upregulated *LoTPS* promoted trehalose synthesis and accumulation to protect adults from low-temperature damage. To further verify the function of the *LoTPS* gene, we employed RNA interference (RNAi) technology. Our findings showed that RCH efficiency disappeared and the survival rate did not increase when the adults were fed dsRNA of *LoTPS*. Additionally, inhibiting *LoTPS* expression resulted in no significant difference in trehalose content between the RCH and non-RCH treatments. Furthermore, the expression patterns of trehalose transporter (*TRET*) and trehalase (*TRE*) were also affected. Collectively, these results indicate the critical role of *LoTPS* in *L. oryzophilus* cold resistance after RCH induction. *LoTPS* can enhance survival ability by regulating trehalose metabolism. These findings contribute to further understanding the role of *TPS* in insect cold resistance and the invasiveness of *L. oryzophilus*. Moreover, RNAi of *LoTPS* opens up possibilities for novel control strategies against *L. oryzophilus* in the future.

## 1. Introduction

Insects have developed various physiological mechanisms to cope with the detrimental effects of low temperatures. Among these, rapid cold hardening (RCH) is one of the adaptive responses in insects [1]. RCH is a type of phenotypic plasticity that allows ectotherms to quickly enhance cold tolerance in response to brief chilling (lasting minutes to hours). RCH protects against nonlethal cold injury by preserving essential functions following cold stress, such as locomotion, reproduction, and energy balance. RCH occurs following a brief exposure to milder cold temperatures and provides rapid protection against sudden drops in temperature [2]. During spring and autumn, RCH can significantly enhance the survival rates of insects, particularly when temperatures are prone to fluctuations or sharp declines. Previous studies have demonstrated increased survival rates under low temperatures following RCH in different insect species, including *Agasicles hygrophila*, *Helicoverpa assulta*, *Solenopsis invicta*, and *Chilo suppressalis* [3,4,5,6].

Recent advances in metabolomics have confirmed the significance of low-molecular-weight sugars and polyols as crucial metabolites produced during RCH in many insect species. Although different insects accumulate different types of cryoprotectants such as subitol, glucose, glycerol, and trehalose [7,8,9,10], trehalose has been implicated in RCH. Several studies have provided evidence linking increased trehalose levels to enhanced cold tolerance through RCH [9,10,11,12,13]. Specifically, trehalose accumulation in the hemolymph stabilizes proteins and maintains cell membrane integrity during RCH [2,8,14].

Trehalose is predominantly synthesized in the fat body in insects [15]. It is then transported to the hemolymph and further to other tissues by trehalose transporters (TRET) [16]. Eventually, trehalose is broken down into two glucose moieties by the trehalase enzyme (TRE). Among the reported trehalose biosynthesis pathways in insects, trehalose is mainly synthesized by the trehalose-6-phosphate synthase (TPS)/trehalose-6-phosphate phosphatase (TPP) pathway or TPS pathway [16]. In the TPS/TPP pathway, TPS transfers glucose from UDP-glucose to glucose-6-phosphate, yielding trehalose-6-phosphate and UDP. TPP converts trehalose-6-phosphate to trehalose. In the TPS pathway, the *TPS* gene is a fused gene encoding proteins with both TPS and TPP domains and exhibits both TPS and TPP enzyme activities [17,18]. *TPS* genes have been identified from different insect species [19,20,21,22,23,24]. However, *TPP* genes have only been found in certain insect species, predominantly in dipteran insects [25,26,27]. Three categories of *TPS* genes (*TPS1*, *TPS2*, and *TPS3*) have been discovered in insects to date [28,29]. Some insects have a single *TPS* gene [18,24,28,30], while others have two [31,32] or three *TPS* genes [29,33]. Interestingly, certain insects such as *Drosophila melanogaster*, *Delia antiqua*, and *Plutella xylostella* also have distinct *TPS* and *TPP* genes [34,35,36,37]. However, the mode and functioning of *TPS* genes in *Lissorhoptrus oryzophilus* Kuschel (Coleoptera: Curculionidae) have not been reported.

The rice water weevil (RWW) is the most important invasive pest of rice (*Oryza sativa* L.) in China. It was initially introduced from Korea in 1988 and has since expanded its distribution to twenty-five provinces, with a continuing trend towards both the northern and southern regions of China. Due to its significant impact on agriculture, it has been designated a national agricultural plant quarantine pest. The adult weevils feed on mesophyll, leading to distinctive longitudinal scars along the leaf blade [20,38]. However, the larvae cause more severe damage by feeding on the roots. Rice water weevils cause yield losses of up to 25% in the absence of prevention [39,40]. In Northeast China, *L. oryzophilus* completes only one generation per year, with adult weevils entering diapause and overwintering at the base of perennial grasses on field bunds, levees, field margins, and other uncultivated areas. Studies on the diapause biology of rice water weevils have revealed significant mechanisms of cold hardiness, enabling their survival under relatively cold temperatures during winter. Trehalose has been identified as a major cryoprotectant in overwintering rice water weevils, contributing to their cold tolerance through increased trehalose content [41]. In regions such as Changchun city in northeast China, where temperature fluctuations are common during early spring and autumn, adult weevils may experience sudden temperature drops without prior exposure to acclimation conditions. In such cases, RCH may play a crucial role. However, our understanding of the physiological mechanisms underlying RCH in rice water weevils remains limited.

In this study, we aimed to investigate the role of the *TPS* gene in the cold resistance of *L. oryzophilus*. First, we cloned the *TPS* gene from *L. oryzophilus* adults using transcriptome data and characterized its properties. Next, we examined the potential of the rice water weevil to induce RCH during the adult stage. We analyzed changes in trehalose content and *TPS* gene expression profile in adult weevils after RCH to assess the role of *TPS* in the cold resistance response elicited by RCH. In order to further understand the function of the *TPS* gene in cold resistance, we employed RNAi technology to inhibit its expression in rice water weevil adults. These RNAi-treated weevils were subsequently exposed to low temperatures to evaluate the effectiveness of RCH, and trehalose content and the expression of genes involved in trehalose metabolism were analyzed. These results will provide insight into the regulatory effect of *TPS* on trehalose metabolism in the cold resistance of *L. oryzophilus* and lead to a new potential target for further control of *L. oryzophilus.*

## 2. Materials and Methods

### 2.1. Insects

Adults of *L. oryzophilus* were collected from rice fields near Changchun city, Jilin Province, Northeast China (43°88′ N, 125°35′ E). The collection took place in late May, when the rice seedlings had just been transplanted for approximately one week and the weevils had started their spring feeding. Then, these weevils were transferred to rice seedlings of approximately 20 cm in a plastic box in the laboratory. The conditions for feeding *L. oryzophilus* were as follows: temperature 25 ± 1 °C, photoperiod 16 h/8 h, and relative humidity of 70%.

### 2.2. Low-Temperature Tolerance and Discriminating Temperature Determination

Following the method of Yang et al. (2018) [42], we measured the discriminating temperature to assess the survival of adult *L. oryzophilus* after various pretreatments. The adults were directly transferred from their rearing conditions to a range of subzero temperatures between −2 °C and −12 °C for 2 h in a refrigerator. This was achieved by placing the adults in Petri dishes with a diameter of 10 cm, with each dish containing twenty adults. The control group was exposed to 25 °C for 2 h. To ensure accurate temperature control, a thermometer was placed inside the refrigerator to monitor the temperature, and adjustments were made as necessary prior to each cold-shock treatment. The adults were exposed to subzero temperatures for a duration of 2 h. After the 2 h exposure, the adults were returned to their rearing conditions, and their survival was assessed 2 h later. Adult weevils were considered dead if their legs did not move when lightly touched with a brush.

### 2.3. Induction and Detection of RCH

To assess the efficiency of RCH and determine the conditions for its induction, we followed a modified version of the method described previously [42]. Adult *L. oryzophilus* were placed in Petri dishes with a diameter of 10 cm. We divided the weevils into different groups and subjected them to various temperature treatments. First, the weevils were transferred from their rearing conditions at 25 °C to either 0 °C or 5 °C for different durations (1, 2, 3, or 4 h). Subsequently, they were exposed to the discriminating temperature of −8 °C for 2 h. After this exposure, the weevils were returned to their original rearing conditions at 25 °C, and their survival was assessed after a 2 h recovery period. We considered weevils that were able to move as having survived. For the control group, weevils were directly transferred from room temperature to the discriminating temperature of −8 °C for 2 h. Each treatment group consisted of 20 individuals, and we replicated the experiment five times to ensure reliable results. To further analyze trehalose content and gene expression, all surviving adult weevils were immediately transferred to liquid nitrogen and preserved at −80 °C until further analysis. 

### 2.4. Measurement of Trehalose Content

To determine the trehalose content in the samples, we utilized the Trehalose Content Kit from Solarbio, Beijing, China. Following the instructions provided in the kit, ten adults of *L. oryzophilus* were collected from both the control group and the RCH group. The collected weevils were weighed and transferred into a grinder for grinding. Each group was homogenized with 50 µL of extraction solution and left at room temperature for 45 min to allow for complete extraction. Then, the samples were centrifuged at 8000 rpm for 5 min and cooled to room temperature. Next, 250 µL of the supernatant was mixed with 500 µL of the reaction reagent from the kit. This mixture was incubated in a 95 °C water bath for 10 min to react and release the trehalose. Following incubation, the solution was cooled to room temperature. Finally, the trehalose content was estimated by measuring the absorbance at 620 nm using a spectrophotometer. Three separate biological replicates were performed to ensure the accuracy and reliability of the results.

### 2.5. Sequence Determination and Bioinformatics Analysis of TPS Gene

For the total RNA extraction and cDNA synthesis, we followed the same method as described in a previous study [43]. Total RNA was extracted from the whole bodies of three *L. oryzophilus* adults using RNAiso Plus (Takara, Dalian, China), following the manufacturer’s instructions. The RNA concentration was measured using NanoDrop 2000 (Wilmington, DE, USA) and the integrity was assessed using the RNA Nano 6000 Assay Kit of the Agilent Bio-analyzer 2100 system (Agilent Technologies, Santa Clara, CA, USA). Purified RNA was stored at −80 °C for future experiments. First-strand cDNA synthesis was performed using the PrimeScript^®^ RT reagent kit with gDNA Eraser (TaKaRa, Dalian, China) following the manufacturer’s protocol.

The sequence of *LoTPS* was identified by searching the sequences in our unpublished transcriptome database and annotations for keywords (trehalose-6-phosphatesynthase). The amino acid sequence of putative *LoTPS* was deduced using DNAMAN software. *TPS* conserved domains of the putative *LoTPS* genes were further analyzed using the Pfam database (http://pfam.xfam.org/, accessed on 6 June 2023). Subsequently, the sequence of putative *LoTPS* was verified by PCR amplification reaction and sequencing again. Additionally, we calculated the molecular weight and isoelectric points of the protein using the Compute pI/Mw tool (http://web.expasy.org/compute_pi/, accessed on 6 June 2023). The open reading frames (ORFs) were predicted using the ORF finder (https://www.ncbi.nlm.nih.gov/orffinder/, accessed on 6 June 2023). Furthermore, we predicted signal peptides and transmembrane domains of the putative protein using the SignalP 5.0 server (http://www.cbs.dtu.dk/services/signalP, accessed on 6 June 2023). For multiple sequence alignments of TPS proteins, we employed DNAMAN 6.0 software (Lynnon Corporation, Pointe-Claire, QC, Canada). We compared the deduced amino acid sequence of *TPS* from *L. oryzophilus* with other insect TPS sequences. Finally, we constructed a neighbor-joining phylogenetic tree using MEGA-X 10.0.5 software. This analysis was performed to understand the evolutionary relationships between the TPS proteins from different insect species.

### 2.6. RNAi of TPS

The dsRNA of *LoTPS* was synthesized according to the manufacturer’s recommendations for the T7 RioMAX Express RNAi System (Promega, San Luis Obispo, CA, USA). As a control, green fluorescent protein (GFP) dsRNA was also synthesized. Specific primers for the *LoTPS* dsRNA fragments were designed and synthesized. The primer sequences can be found in Table 1. The synthesized dsRNA was stored at −80 °C.

To prepare the rice leaves for dsRNA feeding experiments, leaves at the jointing stage were selected and cut into strips measuring 5.0 cm × 0.4 cm in size. The two ends of the strips were fixed with clamps and dried in a 40 °C oven for 20 s. The dsRNA was thawed on ice and diluted with RNase-free water to 500 ng/ΜL. Two hundred microliters of diluted dsRNA solution was packed into 10 mL Eppendorf tubes (Eppendorf, Hamburg, Germany). The dried leaf strips were transferred into the Eppendorf tubes after the leaf surface was daubed with dsRNA. The base of the leaf was immersed in dsRNA solution to suck it up. After the leaf strips absorbed the dsRNA for 24 h, they were used to feed the adults of the rice water weevils. The weevils were starved for 48 h before being transferred to the dsRNA-treated leaves. The EP tubes were sealed with gauze, and fresh rice leaves coated with dsRNA were replaced every 24 h. Live adult weevils that had fed for 24 h, 48 h, and 72 h were collected and stored at −80 °C for gene expression analysis. Each treatment contained 20 adults and was repeated 3 times. The adults fed on normal leaves and treated with ds-GFP solution were used as the control.

### 2.7. Expression Analysis Using RT-qPCR

In this experiment, the effect of RCH and RNAi of *LoTPS* on the transcript expression of the genes in trehalose metabolism including the *TPS*, *TRET*, and *TRE* genes was analyzed using qRT-PCR. Total RNA and first-strand cDNA synthesis were performed as described in Section 2.5. Primer pairs for qPCR were designed using Primer 5 software, as shown in Table 1. *RPS18* was used as a reference gene for normalization. For each qPCR, a total volume of 25 µL was prepared. This included 1 µL of template cDNA, 5 µL of SGExcel Fast SYBR Mixture (TransGen, Beijing, China), 0.4 µL of forward primer (10 µm/L), 0.4 µL of reverse primer (10 µm/L), and 18.2 µL of RNase-free water. Quantitative PCR (qPCR) analysis was performed using an ABI 7500 Real-Time PCR System (Applied Biosystems, Carlsbad, CA, USA). The conditions were 95 °C for 3 min, 40 cycles of 95 °C for 10 s, 60 °C for 30 s, and the melt curve construction at 65–95 °C with a 0.5 °C rise per cycle. Every data point was calculated based on three biological replicates and three corresponding technical replicates. *RPS18* was used as the reference gene, and the 2^−ΔΔCt^ method was used to determine the relative expression values.

### 2.8. Rapid Cold Hardening Assay after LoTPS Knockdown by RNAi

To evaluate the effect of knockdown of the *LoTPS* gene on RCH efficiency, test adults were divided into four groups: control with non-RCH (directly transferred to −8 °C for 2 h), control with RCH (exposed to 0 °C for 4 h prior to −8 °C for 2 h), dsTPS with non-RCH (directly transferred to −8 °C for 2 h after knockdown of *TPS*), and dsTPS with RCH (exposed to 0 °C for 4 h prior to −8 °C for 2 h after knockdown of *TPS*). In the RCH bioassay, test adults were transferred to Petri dishes and placed in the refrigerator at the corresponding temperature. After the treatment period, the survival rates of the adults were determined following a 2 h recovery period at 25 °C. The adults were considered alive if they were able to move. Each treatment was replicated five times with 20 adults per treatment. All live adults were transferred directly to liquid nitrogen and placed at −80 °C until trehalose content and gene expression analysis.

### 2.9. Data Analysis

All bioassays were performed with three independent replicates. All statistical tests were carried out with GraphPad Prism 9 software using analysis of variance (ANOVA). Brown–Forsythe and Shapiro–Wilk tests were applied to determine error variance and normality. Survival of *L. oryzophilus* at different temperatures, content of trehalose, and expression level of *LoTPS* after RCH was tested in a one-way ANOVA and Tukey’s post hoc tests. Two-way ANOVA and Tukey’s post hoc tests, with RNAi and treatment as factors, were carried out to assess the effect of gene expression level and content of trehalose after RCH and RNAi. Two-way ANOVA and Tukey’s post hoc tests, with temperature and time as factors, were carried out to assess the effect of RCH on the survival of *L. oryzophilus*. Data are presented as the mean ± standard deviation and differences are considered significant at the *p* < 0.05 level.

## 3. Results

### 3.1. Identification and Characterization of LoTPS

**Figure 1:**
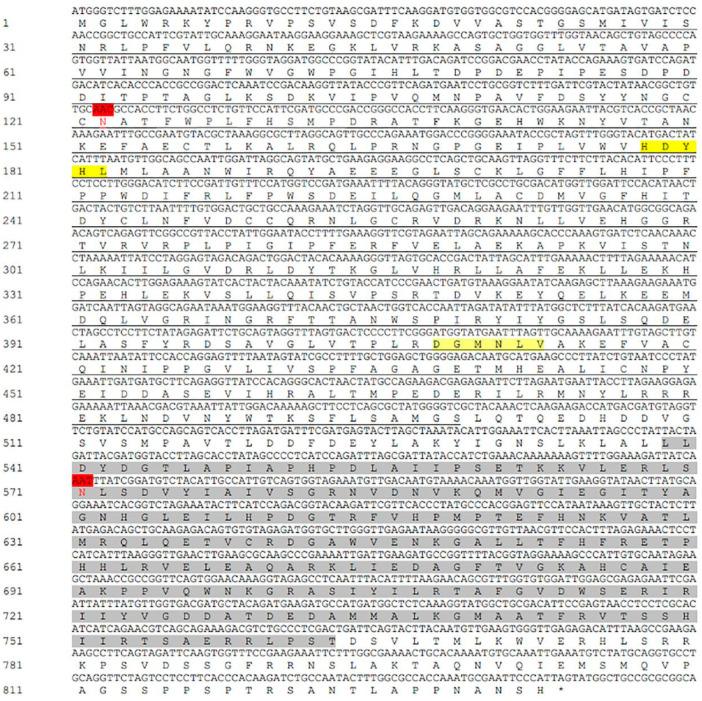
Nucleotide and deduced amino acid sequences of *LoTPS*. The TPS domain is represented by underlined nucleotides, and the TPP domain is shaded in grey. Motifs (or signature motifs) unique to trehalose-6-phosphate synthase (TPS) are shaded in yellow, and predicted N-glycosylation sites are shaded in red.

The sequence of *LoTPS* was identified by searching the sequences in our unpublished transcriptome database and annotations for keywords (trehalose-6-phosphatesynthase). The nucleotide sequence of *LoTPS* is 2499 bp long and encodes a putative protein consisting of 833 amino acids. The cDNA sequence has a theoretical isoelectric point of 6.31. *LoTPS* is a fusion gene that contains two conserved domains: the TPS domain (located at positions 24–474) and the TPP domain (located at positions 539–764). Within the TPS domain, two conserved motifs, HDYHL and DGMNLV, were identified in the *LoTPS* sequence (Figure 1). No signal peptide or transmembrane regions were found in *LoTPS*. There are two potential N-glycosylation sites at positions 122 and 571 (Figure 1).

**Figure 2:**
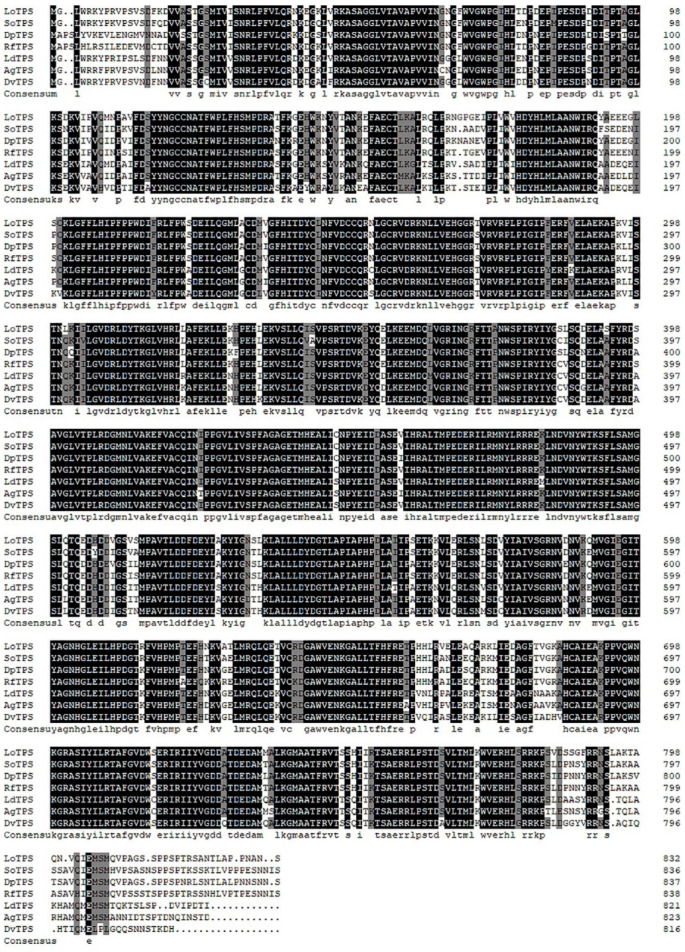
Multiple sequence alignment of trehalose-6-phosphate synthase (TPS) from *L. oryzophilus* with those of other known Coleoptera insects. Amino acid positions with 100% homology are shown in black, those with less than 100% homology and greater than 75% are shown in gray, and those with less than 75% homology are shown in white. So, *Sitophilus oryzae*; (XP_030760030); Dp, *Dendroctonus ponderosae* (XP_019761749.1); Rf, *Rhynchophorus ferrugineus* (KAF7280304.1); Ld, *Leptinotarsa decemlineata* (XP_023020816.1); Aa, *Anoplophora glabripennis* (XP_023311886.1); Dv, *Diabrotica virgifera* (XP_028127759.1).

**Figure 3:**
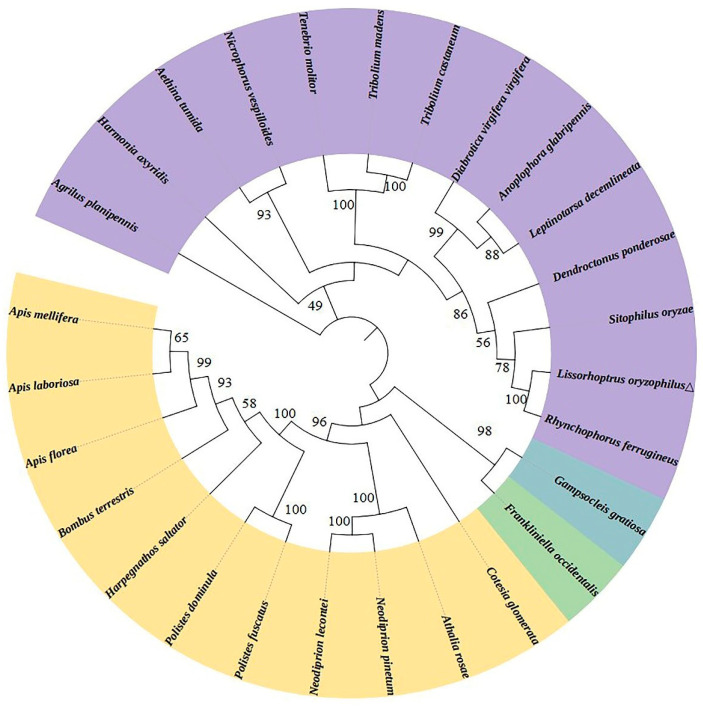
Phylogenetic tree of *LoTPS* genes. The phylogenetic analyses were constructed with the neighbor-joining method. Hymenoptera insects are marked in yellow, Thysanoptera insects are marked in green, Orthoptera insects are marked in blue, and Coleoptera insects are marked in purple. The numbers above the branches represent bootstrap values. The information used to build the tree is shown in the attached Table S1.

The deduced amino acid sequence of *LoTPS* was compared to that of *TPSs* from other species through sequence alignment. The highest similarity was observed with a *TPS* from the Coleoptera species *Rhynchophorus ferrugineus*, with a sequence identity of 93.23%. *LoTPS* also exhibited similarity to *TPSs* from *Dendroctonus ponderosae* (91.45% identity) and *Sitophilus oryzae* (91.08% identity) (Figure 2). The phylogenetic tree analysis further supported the homology among *TPS* genes and their evolutionary relationships with different species. *LoTPS* was found to cluster with other Coleoptera *TPS* genes in the phylogenetic analysis (Figure 3). This suggests that *LoTPS* shares a closer evolutionary relationship with *TPS* genes from other Coleoptera species than with other insect species.

### 3.2. Induction of RCH in L. oryzophilus Adults

**Figure 4:**
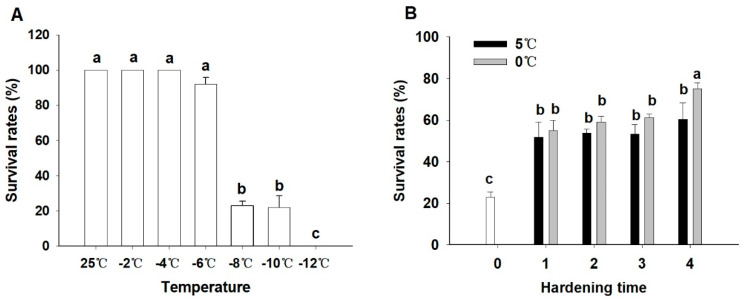
Rapid cold hardening (RCH) of *L. oryzophilus*. (**A**) Discriminating temperature determination. Survival (%, mean ± SD) of *L. oryzophilus* adults after 2 h of exposure to sub-zero temperatures. (**B**) The induction of RCH and the survival of *L. oryzophilus* adults under discriminating temperature by pre-exposure to a cool temperature (0 °C and 5 °C) for 1–4 h. “0” means no pretreatment at cool temperatures and the adults were directly transferred to the discriminating temperature from the rearing temperature (25 °C). For (**A**,**B**), each treatment was replicated five times with 20 adults per replication, bars indicate standard deviation, and means with different letters are significantly different (Tukey’s multiple comparison test at *p* < 0.05).

To determine the discriminating temperature, *L. oryzophilus* adults were directly exposed to sub-zero temperatures for 2 h from the rearing temperature (25 °C). The survival rates of the adults after exposure to temperatures ranging from −2 to −12 °C for 2 h are shown in Figure 4. The results indicated that the survival of adults exposed to temperatures from −2 to −6 °C for 2 h was similar to that of the control (25 °C), at over 90%. However, the survival rates decreased significantly (F = 220.6, *p* < 0.0001), dropping to about 20% at −8 °C and −10 °C, and nearly all adults died after 2 h at −12 °C (Figure 4A). Based on these results, 2 h at −8 °C was chosen as the discriminating temperature.

Then, the RCH response and the optimal conditions for inducing RCH in *L. oryzophilus* adults were determined. The results indicated that pre-exposure to 0 °C or 5 °C over 1 to 4 h (RCH) significantly elevated cold tolerance of adults against −8 °C for 2 h (F = 26.44, *p* < 0.0001). The survival rates at the discriminating temperature were approximately twice as high for the groups pre-treated at 5 °C or 0 °C for 1 to 4 h compared to those with no pre-treatment. Additionally, we found a significantly higher survival rate for the 4 h duration at 0 °C compared to 5 °C (Figure 4B). Based on these findings, an exposure of 4 h at 0 °C was selected as the condition for inducing RCH in the subsequent experiments.

### 3.3. Roles of LoTPS in Trehalose Biosynthesis during RCH

**Figure 5:**
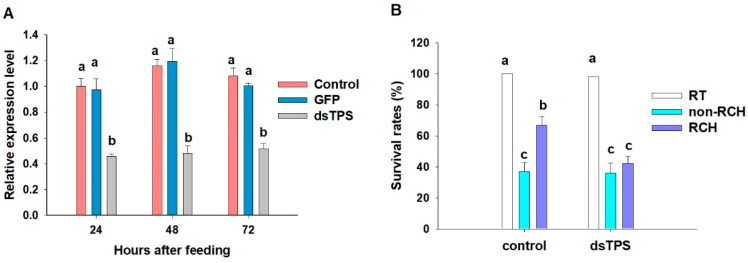
Suppression of *LoTPS* via RNA interference (**A**) and the effect of *LoTPS* RNAi on survival rate after RCH treatment (**B**). (**A**) The relative expression level of *LoTPS* in adults was examined via quantitative qRT-PCR. Control represents the adults feeding on normal leaves without dsRNA. Error bars represent the standard deviation of the calculated means based on three biological replicates. Different letters above the error bars indicate significant differences between treatments and the control measured at the same time (*p* < 0.05). (**B**) The survival rates after suppression of *LoTPS* genes via RNAi and RCH induction at low temperature. RT indicates room temperature. Each treatment was replicated three times with 20 adults per replication. Different letters indicate a significant difference (*p* < 0.05).

To investigate the role of *LoTPS* during RCH, we first fed adult *L. oryzophilus* individuals with rice leaves containing *dsLoTPS*, aiming to suppress the expression of *LoTPS*. Quantitative real-time PCR was used to measure the expression of *LoTPS* 24, 48, or 72 h after exposure to *LoTPS* dsRNA. The results showed that the expression of *LoTPS* was significantly down-regulated compared to that in the control and GFP treatment (F = 247.3, *p* < 0.0001). The interference efficiency did not show a significant difference among the three durations (F = 3.477, *p* = 0.0529) (Figure 5A). Therefore, we have chosen the duration of 24 h as the optimal time for *dsLoTPS* treatment.

Then, the effect of *LoTPS* suppression on RCH efficiency was examined; RNAi-treated adults were exposed to RCH conditions followed by the discriminating temperatures. The survival rate of the control with RCH induction was over 60%, while less than 40% of the control group with non-RCH individuals survived when adults were exposed to the discriminating temperatures. RCH induction significantly increased the survival rate in the RCH group (F = 12.68, *p* < 0.0001). However, there was no RCH effect observed in the adults fed with *dsLoTPS*. Even when the adults were pre-exposed to RCH (*dsLoTPS* with RCH), the survival rate at the discriminating temperatures did not increase significantly compared to that of the non-RCH group (F = 2.639, *p* = 0.4457) (Figure 5B).

**Figure 6:**
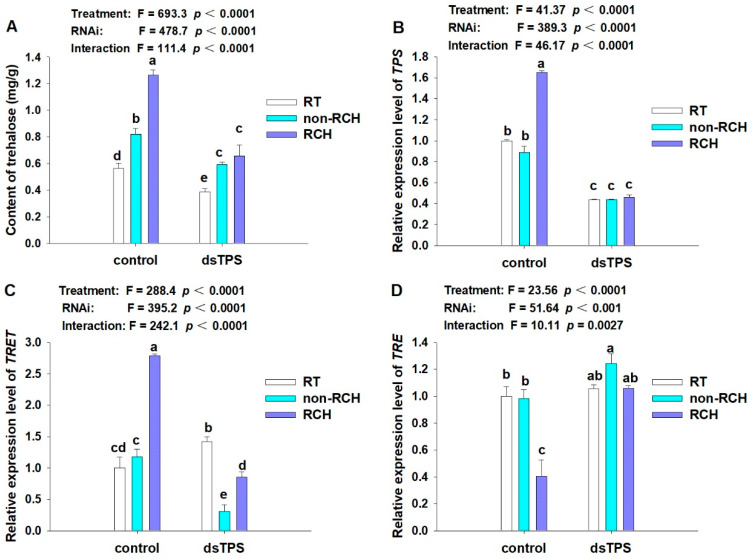
Effect of *LoTPS* RNAi on the content of trehalose (**A**) and expression levels of key genes related to trehalose biosynthesis after RCH induction including *TPS* (**B**), *TRET* (**C**), and *TRE* (**D**) in *L. oryzophilus*. Control represents the adults feeding on normal leaves without dsRNA. Error bars represent the standard error of the calculated means based on three biological replicates. Different letters on the error bars indicate significant differences (*p* < 0.05).

Next, the trehalose content and the relative expression level of the *LoTPS* gene were determined to understand the mechanism by which *TPS* regulates trehalose levels after RCH induction, as shown in Figure 6A,B. It was observed that the suppression of *LoTPS* expression through RNAi significantly inhibited RCH and trehalose accumulation (F = 334.7, *p* < 0.0001) (Figure 6A). There was no significant difference in trehalose content between the non-RCH and RCH treatments in adults fed on *dsLoTPS* (F = 4.106, *p* = 0.074). In contrast, effective RCH in *L. oryzophilus* was accompanied by trehalose accumulation in the control group. Under low temperatures, the amount of trehalose was altered significantly, with an increase observed after exposure to −8 °C for 2 h, both in the treatment pre-exposed to 0 °C for 4 h (RCH) and that with no pre-exposure (non-RCH) (F = 320.2, *p* < 0.0001). The RCH treatment exhibited the highest trehalose content level compared to non-RCH and control treatments.

Gene expression analysis showed that the expression of *LoTPS* at discriminating temperature significantly increased in the RCH treatment group (F = 41.37, *p* < 0.0001). There was no significant difference in expression between the non-RCH and control groups (Figure 6B). However, the relative expression level did not increase in the RCH and non-RCH group adults when the *LoTPS* expression was reduced by specific dsRNA. This indicates that the *LoTPS* gene is upregulated and involved in regulating trehalose synthesis after RCH to enhance the cold resistance of weevil adults.

Finally, the expression levels of the other two key genes associated with trehalose mechanism, *TRE* and *TRET*, were measured with qRT-PCR. Compared to non-RCH treatment, the expression of the *TRET* gene was found to be upregulated in *L. oryzophilus* adults under low temperatures after RCH induction in both control and *dsTPS* treatments. It showed a significant decrease in *TRET* gene expression in non-RCH and RCH group after *LoTPS* dsRNA treatment (F = 288.4, *p* < 0.0001) (Figure 6C). On the other hand, the expression of the *TRE* gene was significantly decreased after RCH in the control group (F = 51.64, *p* < 0.001), but there was no significant difference between the RCH and non-RCH adults when *LoTPS* was suppressed (F = 0.940, *p* = 0.1157) (Figure 6D).

## 4. Discussion

In the TPP/TPS pathway of trehalose synthesis of insects, the two key enzymes involved are TPS and TPP [21]. In our study, the *TPS* gene was identified from the transcriptome of *L. oryzophilus* adults and designated as *LoTPS*. Similar to other known *TPS* genes in insects [28,34,44], the *LoTPS* gene is also a fused gene. The deduced *LoTPS* amino acid sequence encodes two functional domains, an N-terminal TPS domain and a C-terminal TPP domain (one Glyco_transf_20 domain and one Trehalose_Ppase domain), and contains two signature motifs (HDYHL and DGMNLV). Through multiple sequence alignment, we found that the deduced amino acid sequence of *LoTPS* exhibits a high level of identity with homologous proteins reported in other Coleoptera insects, specifically *R. ferrugineus* and *D. ponderosae*, with a sequence identity of over 91%. Furthermore, the phylogenetic tree clearly indicates that TPS from different insects can be grouped into four major clades. As expected, *LoTPS* clusters together with TPS proteins from Coleoptera species, indicating a close evolutionary relationship between *LoTPS* and its homologues in Coleoptera insects.

RCH can offer protection in insects against acute cold stress [45]. In our study, we found that RCH treatment for 4 h at 0 °C significantly increased the cold tolerance capabilities of *L. oryzophilus* adults. This was evident by the increased survival rate when they were exposed to a discriminating temperature of −8 °C. In addition to *L. oryzophilus*, RCH response has been observed in many insects such as *H. assulta*, *Trogoderma granarium, Spodoptera frugiperda*, and *Liriomyza sativae*, and prevents chilling injury by pre-exposing insects to nonlethal low temperatures [5,8,46,47]. Therefore, the ability of RCH is beneficial and essential for *L. oryzophilus* to cope with thermally variable environments.

Cold tolerance in insects is a complex adaptive response that involves significant changes in biochemistry, gene expression, cell function, and the endocrinological system. These adaptations allow for increased cell function and viability at low temperatures [2]. In our study, a high level of trehalose was observed in the RCH group. As a major cryoprotectant, the accumulation of trehalose plays a physiological role in the capacity of insects to survive freezing [44,48,49]. Trehalose is reported to play a role in the stabilization of phospholipids in the plasma membrane, which is a target for cold-induced injury [7]. Increased trehalose content in response to cold acclimation has also been reported in diapausing *L. oryzophilus* populations [41]. These results suggest that *L. oryzophilus* can use trehalose as a cryoprotectant to resist cold shock under low temperatures. Similarly, RCH also induced the accumulation of trehalose in *Thrips palmi* and *Drosophila melanogaster* [11,50]. However, Overgaard et al. (2014) and Mohammadzadeh (2018) found that RCH had no significant effects on trehalose [8,51]. This suggests that insects may become cold-hardy due to RCH if RCH participates in the accumulation of cryoprotectants.

We hypothesized that changes in trehalose concentrations may be accompanied by alterations in the expression of genes associated with trehalose metabolism. In insects, trehalose synthesis is triggered by TPS [52]. In our study, the *LoTPS* gene showed upregulated expression during RCH, which may be relevant to the accumulation of trehalose. Trehalose biosynthesis genes have been observed to be upregulated following RCH induction in various insect species. Earlier studies on *Maruca vitrata* and *P. xylostella* showed that RCH increased the expression of *TPS* and trehalose concentration [5,13,53]. These findings support the idea that TPS is an essential factor contributing to RCH-induced cold tolerance. We found that trehalose levels were elevated in the non-RCH samples, although the relative expression level of *LoTPS* was not increased compared to the control. We hypothesized that the possible reasons for this observation was that only one *LoTPS* gene was cloned and determined, and that there are other *TPS* genes in rice water weevil that regulate trehalose synthesis at low temperatures. On the other hand, the trehalose content may be affected by trehalose transporter and trehalase. In insects, the trehalose transporter TRET1 was found to be involved in transporting trehalose synthesized in the fat body into the hemolymph and regulating the trehalose content in different tissues [54]. In our study, RCH treatment increased the expression level of TRET in *L. oryzophilus* adults, which may regulate the distribution and balance of trehalose in different tissues in order to improve the cold resistance of adults, but this requires further study. It has been demonstrated that TRET1a is highly expressed in the fat bodies of diapause-destined *Colaphellus bowringi* and regulates trehalose out of the fat body [55]. Trehalase is the only enzyme currently known to be capable of breaking down trehalose. In *L. oryzophilus*, we have cloned one soluble trehalase, *LoTRE1* [43]. In contrast, *TRE* expression is significantly down-regulated in response to RCH, indicating that the *TRE* of *L. oryzophilus* is most likely responsible for regulating trehalose degradation, contributing to trehalose accumulation.

Furthermore, we confirmed the importance of *LoTPS* in low temperature tolerance after RCH through RNA interference. The results showed that *LoTPS* expression was effectively suppressed by RNAi at 24 h post-feeding specific *dsRNA* against *LoTPS*. Accordingly, the survival rates of the *dsRNA*-treated group were not improved at low temperature after RCH induction compared to those of the control group. This suggests that the suppression of *LoTPS* expression had a negative impact on the ability of *L. oryzophilus* adults to survive at low temperatures after RCH induction. Similar findings were observed in *P. xylostella*, the suppression of *PxTPS* via RNA interference decreased survival rate under RCH [52]. These results have further verified the importance of *TPS* genes. Their involvement in cold resistance has been well defined [45,56]. In addition, the trehalose content in the adults decreased significantly after RNAi. These findings confirm that the suppression of *LoTPS* by RNAi inhibits trehalose biosynthesis and disrupts the protective effect of RCH. The silencing of *LoTPS* genes not only reduced the expression level of the target gene, but also affected that of the *TRET* and *TRE* genes. Specifically, the expression of the *TRET* gene did not increase under low temperature after *TPS* gene interference compared to RT treatment. But its expression of RCH treatment was significantly higher than that of non-RCH treatment in both the control and dsTPS groups. For *TRE*, RCH significantly reduced its expression at low temperatures, but there was no significant difference between the RT, non-RCH and RCH groups after RNA interference. These results suggest that the coordination of trehalose synthesis, transportation, and degradation pathways is responsible for trehalose accumulation with RCH. We speculate that RNAi can effectively suppress the expression of *LoTPS*, thus disrupting trehalose metabolism and affecting the cold tolerance of *L. oryzophilus* adults. However, the molecular mechanisms underlying trehalose accumulation in this context require further study.

## 5. Conclusions

In conclusion, our study has demonstrated that rapid cold hardening (RCH) enhances the survival of *L. oryzophilus* adults at low temperatures. This improvement in survival is attributed to the upregulation of *LoTPS* expression, which leads to increased trehalose biosynthesis. However, when *LoTPS* was suppressed through RNAi, the efficiency of RCH at low temperatures disappeared, indicating the essential role of *LoTPS* in trehalose biosynthesis. These findings highlight the significance of trehalose as a cryoprotectant for acquiring cold hardiness in *L. oryzophilus*, with *LoTPS* serving as a critical regulator of trehalose biosynthesis.

## Figures and Tables

**Table 1:** Primer sequences used in this study.

Primer Name	Primer Sequence (5′-3′)	Purpose
*LoTPS*	GCGTTTGGTGTGGATTGG/ATACGCTGACATCACCCC	ORF cloning
*LoTPS*	GCGTTTGGTGTGGATTGG/GATGATGTGCGAGGAGGT	RT-qPCR
*TRET*	ACCACGACTCAGGAAAAT/ACCAACGCATAAGATAGC	
*TRE*	AACCTGTGATTGTCCCTG/TCCTTTGGCTGTTTCGTG	
*RpS18*	GTAATGTTTGCCTTGACTG/TTTCTACTTCCTCTTCGG	
*dsTPS*-F	TAATACGACTCACTATAGGGGACAAAAAGCTTCCTCAGCG	RNAi
*dsTPS*-R *dsGFP*-F	TAATACGACTCACTATAGGGAGTGGAACGTTAACAACGCC	
	TAATACGACTCACTATAGGGTGTTCTGCTGGTAGTGGTCG	
*dsGFP*-R	TAATACGACTCACTATAGGGTGTTCTGCTGGTAGTGGTCG	

## Data Availability

The data presented in this study are available in the article.

## Supplementary Materials

The following supporting information can be downloaded at: https://www.mdpi.com/article/10.3390/insects14120903/s1, Table S1: GeneBank accession number of species and gene sequences to construct LoTPS phylogenetic tree.

## References

[1] Lee R.E., Chen C.P., Denlinger D.L. (1987). A Rapid Cold-Hardening Process in Insects. Science.

[2] Teets N.M., Denlinger D.L. (2013). Physiological Mechanisms of Seasonal and Rapid Cold-Hardening in Insects. Physiol. Entomol..

[3] Yang G., Wen J., Han Y., Hou M. (2018). Rapid Cold Hardening Confers a Transient Increase in Low Temperature Survival in Diapausing *Chilo suppressalis* Larvae. Insects.

[4] Vatanparast M., Sajjadian S.M., Park Y. (2022). Glycerol Biosynthesis Plays an Essential Role in Mediating Cold Tolerance the Red Imported Fire Ant, *Solenopsis invicta*. Arch. Insect Biochem. Physiol..

[5] Cha W.H., Lee D. (2016). Identification of Rapid Cold Hardening-Related Genes in the Tobacco Budworm, *Helicoverpa assulta*. J. Asia-Pac. Entomol..

[6] Pei Y., Jin J., Wu Q., Liang X., Lv C., Guo J. (2023). Cold Acclimation and Supercooling Capacity of *Agasicles aygrophila* Adults. Insects.

[7] Michaud M.R., Denlinger D.L. (2007). Shifts in the Carbohydrate, Polyol, and Amino Acid Pools during Rapid Cold-Hardening and Diapause-Associated Cold-Hardening in Flesh Flies (*Sarcophaga crassipalpis*): A Metabolomic Comparison. J. Comp. Physiol. B.

[8] Mohammadzadeh M., Izadi H. (2018). Cold Acclimation of *Trogoderma granarium* Everts Is Tightly Linked to Regulation of Enzyme Activity, Energy Content, and Ion Concentration. Front. Physiol..

[9] Izadi H., Mohammadzadeh M., Mehrabian M. (2019). Cold Tolerance of the *Tribolium castaneum* (Coleoptera: Tenebrionidae), Under Different Thermal Regimes: Impact of Cold Acclimation. J. Econ. Entomol..

[10] Xie Z., Xu L., Zhao J., Li N., Qin D., Xiao C., Lu Y., Guo Z. (2023). Rapid Cold Hardening and Cold Acclimation Promote Cold Tolerance of Oriental Fruit Fly, *Bactrocera dorsalis* (Hendel) by Physiological Substances Transformation and Cryoprotectants Accumulation. Bull. Entomol. Res..

[11] Overgaard J., Malmendal A., Sørensen J.G., Bundy J.G., Loeschcke V., Nielsen N.C., Holmstrup M. (2007). Metabolomic Profiling of Rapid Cold Hardening and Cold Shock in *Drosophila* melanogaster. J. Insect Physiol..

[12] Zheng X.L., Zhou L.J., Lu W., Xian Z.H., Yang Z.D., Lei C.L., Wang X.P. (2014). Cold-Hardiness Mechanisms in Third Instar Larvae of *Spodoptera exigua* Hübner (Lepidoptera: Noctuidae). Afr. Entomol..

[13] Kim Y., Lee D.W., Jung J.K. (2017). Rapid Cold-Hardening of a Subtropical Species, *Maruca vitrata* (Lepidoptera: Crambidae), Accompanies Hypertrehalosemia by Upregulating Trehalose-6-Phosphate Synthase. Environ. Entomol..

[14] Koštál V., Korbelová J., Rozsypal J., Zahradníčková H., Cimlová J., Tomčala A., Šimek P. (2011). Long-Term Cold Acclimation Extends Survival Time at 0℃ and Modifies the Metabolomic Profiles of the Larvae of the Fruit Fly *Drosophila melanogaster*. PLoS ONE.

[15] Arrese E.L., Soulages J.L. (2010). Insect Fat Body: Energy, Metabolism, and Regulation. Annu. Rev. Entomol..

[16] Mitsumasu K., Kanamori Y., Fujita M., Iwata K., Tanaka D., Kikuta S., Watanabe M., Cornette R., Okuda T., Kikawada T. (2010). Enzymatic Control of Anhydrobiosis-Related Accumulation of Trehalose in the Sleeping Chironomid, *Polypedilum vanderplanki*. FEBS J..

[17] Chen Q., Ma E., Behar K.L., Xu T., Haddad G.G. (2002). Role of Trehalose Phosphate Synthase in Anoxia Tolerance and Development in *Drosophila* melanogaster. J. Biol. Chem..

[18] Jin T.T., Gao Y.L., He K.L., Ge F. (2018). Expression Profiles of the Trehalose-6-Phosphate Synthase Gene Associated with Thermal Stress in *Ostrinia furnacalis* (Lepidoptera: Crambidae). J. Insect Sci..

[19] Yang M., Zhao L., Shen Q., Xie G., Wang S., Tang B. (2017). Knockdown of Two Trehalose-6-Phosphate Synthases Severely Affects Chitin Metabolism Gene Expression in the Brown Planthopper *Nilaparvata lugens*. Pest Manag. Sci..

[20] Shi J.F., Xu Q.Y., Sun Q.K., Meng Q.W., Mu L.L., Guo W.C., Li G.Q. (2016). Physiological Roles of Trehalose in Leptinotarsa Larvae Revealed by RNA Interference of Trehalose-6-Phosphate Synthase and Trehalase Genes. Insect Biochem. Mol. Biol..

[21] Chen J.X., Lyu Z.H., Wang C.Y., Cheng J., Lin T. (2020). RNA Interference of a Trehalose-6-Phosphate Synthase Gene Reveals Its Roles in the Biosynthesis of Chitin and Lipids in *Heortia vitessoides* (Lepidoptera: Crambidae). Insect Sci..

[22] Zhang H.Z., Wang M.Q., Xie Y.Q., Xiang M., Li P., Li Y.Y., Zhang L.S. (2020). Gene Cloning and Expression Analysis of Trehalose-6-Phosphate Synthase, Glycogen Synthase and Glycogen Phosphorylase Reveal the Glycometabolism in the Diapause Process of *Aphidius gifuensis*. J. Asia-Pac. Entomol..

[23] Zhang Y., Wang F., Feng Q., Wang H., Tang T., Huang D., Liu F. (2019). Involvement of Trehalose-6-Phosphate Synthase in Innate Immunity of *Musca domestica*. Dev. Comp. Immunol..

[24] Yang H.J., Cui M.Y., Zhao X.H., Zhang C.Y., Hu Y.S., Fan D. (2023). Trehalose-6-Phosphate Synthase Regulates Chitin Synthesis in *Mythimna separate*. Front. Physiol..

[25] Kushwaha S., Singh P.K., Shahab M., Pathak M., Bhattacharya S.M. (2012). *In Vitro* Silencing of *Brugia Malayi* Trehalose-6-Phosphate Phosphatase Impairs Embryogenesis and In Vivo Development of Infective Larvae in Jirds. PLoS Negl. Trop. Dis..

[26] Yoshida M., Matsuda H., Kubo H., Nishimura T. (2016). Molecular Characterization of *Tps1* and *Treh* Genes in Drosophila and Their Role in Body Water Homeostasis. Sci. Rep..

[27] Wang J., Fan H., Li Y., Zhang T.F., Liu Y.H. (2022). Trehalose-6-Phosphate Phosphatases Are Involved in Trehalose Synthesis and Metamorphosis in *Bactrocera minax*. Insect Sci..

[28] Tang B., Wang S., Wang S.G., Wang H.J., Zhang J.Y., Cui S.Y. (2018). Invertebrate Trehalose-6-Phosphate Synthase Gene: Genetic Architecture, Biochemistry, Physiological Function, and Potential Applications. Front. Physiol..

[29] Wang S.S., Li G.Y., Liu Y.K., Luo Y.J., Xu C.D., Li C., Tang B. (2020). Regulation of Carbohydrate Metabolism by Trehalose-6-Phosphate Synthase 3 in the Brown Planthopper, *Nilaparvata lugens*. Front. Physiol..

[30] Gong C., Yang Z., Hu Y., Wu Q., Wang S., Guo Z., Zhang Y. (2022). Silencing of the *BtTPS* Genes by Transgenic Plant-Mediated RNAi to Control *Bemisia tabaci* MED. Pest Manag. Sci..

[31] Chen Q.W., Jin S., Zhang L., Shen Q.D., Wei P., Wei Z.M., Wang S.G., Tang B. (2018). Regulatory Functions of Trehalose-6-phosphate Synthase in the Chitin Biosynthesis Pathway in *Tribolium castaneum* (Coleoptera: Tenebrionidae) Revealed by RNA Interference. Bull. Entomol. Res..

[32] Ding Y.J., Li G.Y., Xu C.D., Wu Y., Zhou Z.S., Wang S.G., Li C. (2020). Regulatory Functions of *Nilaparvata lugens* GSK-3 in Energy and Chitin Metabolism. Front. Physiol..

[33] Zhou M., Shen Q., Wang S.S., Li G.Y., Wu Y., Xu C.D., Tang B., Li C. (2022). Regulatory Function of The Trehalose-6-Phosphate Synthase Gene *TPS3* on Chitin Metabolism in Brown Planthopper, *Nilaparvata lugens*. Insect Mol. Biol..

[34] Cui S.Y., Xia Y.X. (2009). Isolation and Characterization of the Trehalose-6-Phosphate Synthase Gene from Locusta *migratoria manilensis*. Insect Sci..

[35] Huang Q., Zhang G., Nan J., Cheng W., Zhu-Salzman K. (2021). Characterization of Trehalose Metabolic Genes and Corresponding Enzymatic Activities during Diapause of *Sitodiplosis mosellana*. J. Insect Physiol..

[36] Guo Q., Hao Y.-J., Li Y., Zhang Y.-J., Ren S., Si F.-L., Chen B. (2015). Gene Cloning, Characterization and Expression and Enzymatic Activities Related to Trehalose Metabolism during Diapause of the Onion Maggot *Delia antiqua* (Diptera: Anthomyiidae). Gene.

[37] Dmitryjuk M., Łopieńska-Biernat E., Zaobidna E.A. (2014). The *In Vitro* Effect of Ivermectin on the Activity of Trehalose Synthesis Pathway Enzymes and Their mRNA Expression in the Muscle of Adult Female *Ascaris suum* (Nematoda). Sci. World J..

[38] Stout M.J., Rice W.C., Ring D.R. (2002). The Influence of Plant Age on Tolerance of Rice to Injury by the Rice Water Weevil, *Lissorhoptrus oryzophilus* (Coleoptera: Curculionidae). Bull. Entomol. Res..

[39] Reay-Jones F.P.F., Way M.O., Tarpley L. (2008). Nitrogen Fertilization at the Rice Panicle Differentiation Stage to Compensate for Rice Water Weevil (Coleoptera: Curculionidae) Injury. Crop Prot..

[40] Aghaee M., Godfrey L.D. (2014). A Century of Rice Water Weevil (Coleoptera: Curculionidae): A History of Research and Management with an Emphasis on the United States. J. Integr. Pest Manag..

[41] Lee K.Y., Chang Y.D., Kim Y.G. (2002). Trehalose, A Major Sugar Cryoprotectant of the Overwintering Rice Water Weevil, *Lissorhoptrus oryzophilus* (Coleoptera: Curculionidae). J. Asia-Pac. Entomol..

[42] Yang S., Zhang X.X., Wang J.X., Wang S., Pan Y., Zhang J.H., Xi J.H. (2018). Identification and Analysis of Up-Regulated Proteins in *Lissorhoptrus oryzophilus* Adults for Rapid Cold Hardening. Gene.

[43] Wang Q., Fang K., Qi L., Wang X., Pan Y., Li Y., Xi J., Zhang J. (2022). Purification and Functional Characterization of a Soluble Trehalase in *Lissorhoptrus oryzophilus* (Coleoptera: Curculionidae). Insects.

[44] Lü X., Han S., Li Z., Li L., Li J. (2020). Gene Characterization and Enzymatic Activities Related to Trehalose Metabolism of In Vitro Reared *Trichogramma dendrolimi* Matsumura (Hymenoptera: Trichogrammatidae) under Sustained Cold Stress. Insects.

[45] Teets N.M., Gantz J.D., Kawarasaki Y. (2020). Rapid Cold Hardening: Ecological Relevance, Physiological Mechanisms and New Perspectives. J. Exp. Biol..

[46] Malekera M.J., Acharya R., Hwang H.S., Lee K.Y. (2022). Effect of Cold Acclimation and Rapid Cold-Hardening on the Survival of *Spodoptera frugiperda* (J.E. Smith) (Lepidoptera: Noctuidae) under Cold Stress. J. Asia-Pac. Entomol..

[47] Iqbal J., Zhang X.-X., Chang Y.-W., Du Y.-Z. (2021). Differential Response of Leafminer Flies *Liriomyza trifolii* (Burgess) and *Liriomyza sativae* (Blanchard) to Rapid Cold Hardening. Insects.

[48] Tamang A.M., Kalra B., Parkash R. (2017). Cold and Desiccation Stress Induced Changes in the Accumulation and Utilization of Proline and Trehalose in Seasonal Populations of *Drosophila immigrans*. Comp. Biochem. Physiol. A Mol. Integr. Physiol..

[49] Yu C., Zhao R., Zhou W., Pan Y., Tian H., Yin Z., Chen W. (2022). Fruit Fly in a Challenging Environment: Impact of Short-Term Temperature Stress on the Survival, Development, Reproduction, and Trehalose Metabolism of *Bactrocera dorsalis* (Diptera: Tephritidae). Insects.

[50] Park K., Kim K., Kim Y. (2014). Rapid Cold hardening of Thrips palmi (Thysanoptera: Thripidae). Environ. Entomol..

[51] Overgaard J., Sorensen J.G., Com E., Colinet H. (2014). The rapid cold hardening response of Drosophila melanogaster: Complex regulation across different levels of biological organization. J. Insect Physiol..

[52] Tellis M.B., Kotkar H.M., Joshi R.S. (2023). Regulation of Trehalose Metabolism in Insects: From Genes to the Metabolite Window. Glycobiology.

[53] Cha W.H., Lee D.W. (2018). RNA Interference of Trehalose Phosphate Synthase Inhibits Metamorphosis and Decreases Cold Tolerance in the Diamondback Moth, *Plutella xylostella* (L.). J. Asia-Pac. Entomol..

[54] Zhou H., Lei G., Chen Y., You M., You S. (2022). PxTret1-like Affects the Temperature Adaptability of a Cosmopolitan Pest by Altering Trehalose Tissue Distribution. Int. J. Mol. Sci..

[55] Li J.X., Cao Z., Guo S., Tian Z., Liu W., Zhu F., Wang X.P. (2020). Molecular characterization and functional analysis of two trehalose transporter genes in the cabbage beetle, Colaphellus bowringi. J. Asia Pac. Entomol..

[56] Wan S., He J., Chao L., Shi Z., Wang S., Yu W., Huang Z., Wang S., Wang S., Zhang Z. (2023). Regulatory Role of Trehalose Metabolism in Cold Stress of *Harmonia axyridis* Laboratory and Overwinter Populations. Agronomy.

