# Supplementary material for: Trehalose-6-Phosphate Synthase Contributes to Rapid Cold Hardening in the Invasive Insect Lissorhoptrus oryzophilus (Coleoptera: Curculionidae) by Regulating Trehalose Metabolism
Source: Insects. 2023 Nov 23;14(12):903. doi: 10.3390/insects14120903 (PMC10744047; doi:10.3390/insects14120903)
Supplement: Supplementary file 1 [file insects-14-00903-s001.zip › insects-2671611-supplementary.pdf]

Table S1. GeneBank accession number of species and gene sequences to construct *LoTPS* phylogenetic tree.

Species	GeneBank Accession Number
Sitophilus oryzae	XP_030760030
Dendroctonus ponderosae	XP_019761749.1
Rhynchophorus ferrugineus	KAF7280304.1
Tenebrio molitor	KAH0815604.1
Tribolium castaneum	XP_975776.2
Leptinotarsa decemlineata	XP_023020816.1
Anoplophora glabripennis	XP_023311886.1
Tribolium madens	XP_044255908.1
Diabrotica virgifera virgifera	XP_028127759.1
Nicrophorus vespilloides	XP_017769007.1
Agrilus planipennis	XP_025832646.1
Aethina tumida	XP_019865342.1
Harmonia axyridis	XP_045480405.1
Frankliniella occidentalis	XP_026272691.1
Cotesia glomerata	XP_044576379.1
Apis mellifera	XP_026297280.1
Gampsocleis gratiosa	APZ77037.1
Polistes dominula	XP_015172546.1
Harpegnathos saltator	XP_011139179.1
Apis laboriosa	XP_043793631.1
Bombus terrestris	XP_020719094.1
Athalia rosae	XP_012252443.1
Polistes fuscatus	XP_043494062.1
Apis florea	XP_031771052.1
Neodiprion lecontei	XP_015522281.2
Neodiprion pinetum	XP_046474850.1
